# More Cancer Cures

**Published:** 1902-02-08

**Authors:** 


### MORE CANCER CURES.

Every week we are told some new story about some new cure for cancer. Even old ones are revived, and if we listen to all that we hear our faith is constantly on the strain. The latest—at the moment of writing—is a revival by Professor Albert Adamkiewicz,[1] of Vienna, of a method which he is said to have originated as far back as 1893. This consists of the injection of "canroin," that is to say of the toxin believed to be produced by the hypothetical micro-organism by the growth of which cancer is imagined to be caused. This way of putting the matter sounds rather like The House that Jack Built, but really it expresses with fair accuracy, so far as positive knowledge goes at the present time, the degree of relationship between the substance called cancroin and the disease which it is intended to cure. To come, however, to Professor Adamkiewicz' experience. The case was one of cancer of the cervix uteri in a woman aged 36 years. It was diagnosed as cancer not only by the practitioner in attendance, but by several specialists whose names are given. The growth was scraped, microscopical examination of the fragments showed its cancerous nature, and the case was regarded as hopeless and quite unfitted for operation. The patient became worse, was unable to leave her bed, pains became very severe, she could take but little food and she vomited what she took. Local examination showed that in the position of the portio vaginalis there was a dense-walled, easily bleeding, cup-shaped excavation, and above the sphincter and on the anterior wall of the rectum a tumour could be felt, of the size and shape of a pear and very painful. The abdomen was extremely painful, and palpation through the walls was impossible. Then the injections of cancroin were commenced. The hæmorrhage and abdominal pains disappeared promptly. On the second day the patient sat up in bed, free from pain and quite at ease, and took her breakfast with relish. She soon had an excellent appetite and all kinds of food agreed with her. Fæces and urine were passed without pain, the tumour which had formed above the sphincter of the rectum diappeared, the excavation of the cervix became smaller and was smooth walled, the patient got up and was apparently quite cured. On giving up the injections, however, many of her symptoms returned and she became very ill again; but again on resuming them, the same wonderful recovery took place. "The metritis gave way as if at the word of command, and the patient began to recover her health," and after four weeks of the resumed treatment the uterus, the fundus of which could not on its upper side be reached from the rectum, was once more able to be palpated, and was again changed into a small, hard, uneven, globular mass, easily defined and no longer painful. Now in regard to all these cures, whether they come from the lips of quacks or of professors, we must be open-eared but cautious-minded. So far as they are true, however fallacious they may be as guides to treatment, they at least are interesting and important facts in regard to the natural history of the disease, or of diseases which are clinically confused with carcinoma. In such a matter as this we have no right to pick and choose our evidence, or to refuse that which comes from outside the profession. The case just mentioned is no doubt a good one, but then so was the case which lately had the run of the papers in which a cancer was said to have been cured by an infusion of violet leaves! In fact this latter, taking it as told, was a very good case indeed; for the cure was complete, the witnesses were above suspicion, the remedy was a simple one accessible to all, and no one concerned in it had any axe to grind. Yet we will undertake to say that every doctor that heard of it said "bosh." When it comes to evidence, however, what is sauce for the goose is sauce for the gander, and when it becomes a question of ascertaining the natural history of cancer all these strange and apparently incredible tales of cure or of recovery —including those by thyroid extract, by oöphorectomy, and by Coley's fluid; the interesting histories of cancers which either under the influence of age, or opium, or other drugs, have become quiescent even without being cured; and also those of cases in which, while one part of a growth has progressed, other parts have, without any known cause, shrivelled and become absorbed. All these curious histories, as well as all the wonderful tales told of recovery from cancer under quack remedies, must be correlated, tested and compared, if we are to arrive at a full and true conception of the influences which make for the spread or encourage the retrogression of this disease.

[1] Lancet, Feb. 1.

